# Virus-Like Particle Systems for Vaccine Development against Viruses in the Flaviviridae Family

**DOI:** 10.3390/vaccines7040123

**Published:** 2019-09-20

**Authors:** Shu Hui Wong, Alagie Jassey, Jonathan Y. Wang, Wei-Cheng Wang, Ching-Hsuan Liu, Liang-Tzung Lin

**Affiliations:** 1 International Master Program in Medicine, College of Medicine, Taipei Medical University, Taipei 11031, Taiwan; m142107001@tmu.edu.tw (S.H.W.); m142107002@tmu.edu.tw (J.Y.W.); 2 Institut de Recherches Cliniques de Montréal, Montreal, QC H2W 1R7, Canada; 3 International Ph.D. Program in Medicine, College of Medicine, Taipei Medical University, Taipei 11031, Taiwan; d142105007@tmu.edu.tw; 4 Graduate Institute of Medical Sciences, College of Medicine, Taipei Medical University, Taipei 11031, Taiwan; m120107011@tmu.edu.tw (W.-C.W.); d119107007@tmu.edu.tw (C.-H.L.); 5 Department of Microbiology & Immunology, Dalhousie University, Halifax, NS B3H 4R2, Canada; 6 Department of Microbiology and Immunology, School of Medicine, College of Medicine, Taipei Medical University, Taipei 11031, Taiwan

**Keywords:** virus-like particles, Flaviviridae, immunogenicity, vaccines

## Abstract

Viruses in the Flaviviridae family are important human and animal pathogens that impose serious threats to global public health. This family of viruses includes emerging and re-emerging viruses, most of which are transmitted by infected mosquito or tick bites. Currently, there is no protective vaccine or effective antiviral treatment against the majority of these viruses, and due to their growing spread, several strategies have been employed to manufacture prophylactic vaccines against these infectious agents including virus-like particle (VLP) subunit vaccines. VLPs are genomeless viral particles that resemble authentic viruses and contain critical repetitive conformational structures on their surface that can trigger the induction of both humoral and cellular responses, making them safe and ideal vaccine candidates against these viruses. In this review, we focus on the potential of the VLP platform in the current vaccine development against the medically important viruses in the Flaviviridae family.

## 1. Introduction

The Flaviviridae family of positive sense, single-stranded enveloped RNA viruses are some of the most widely distributed and important human and animal viral pathogens that cause significant morbidity and mortality worldwide yearly. The most prominent members of this family with epidemic history, emergence/re-emergence, or of medical importance to humans are the focus of this review, and include yellow fever virus (YFV), West Nile virus (WNV), Japanese encephalitis virus (JEV), hepatitis C virus (HCV), dengue virus (DENV), Zika virus (ZIKV), tick-borne encephalitis virus (TBEV), and Powassan virus (POWV). Of which, most are arthropod-borne viruses (arboviruses) transmitted by hematophagous mosquito and tick vectors, with the exception of HCV that is non-arboviral and contracted primarily via blood transmission [1,2]. Infection with these viruses can result in various symptomatic clinical diseases, ranging from non-severe self-limiting flu-like symptoms to severe and potentially life-threatening diseases such as encephalitis and neurological sequelae (for JEV, WNV, ZIKV, TBEV, POWV), jaundice, kidney and liver failure (for YFV), liver damage and cancer (for HCV), and haemorrhagic fever (for DENV) [1,2]. With a general lack of specific antivirals against most of these viral infections, prevention is of paramount importance for reducing the global health threats and disease burden caused by these viruses. Although countermeasures such as the avoidance of mosquito and tick bites, avoidance of infected blood or bodily fluid contact, vector control, and travel precautions are commonly implemented, they have been inefficient due to the reliance on sustained public will and infrastructure [3], as well as uncontrollable factors like climate change and increased travel and trade, which facilitate vector and disease transmission. As such, vaccine development for public immunization remains vital for effective prevention of infection and transmission of these infectious viral diseases.

Belonging to the same family, Flaviviridae, the viruses (YFV, WNV, JEV, HCV, DENV, ZIKV, TBEV, and POWV) share a common genomic organization whereby their RNA genome ranging from 9.6 to 12.3 kb encodes a precursor polyprotein that is post-translationally cleaved into ten proteins—three structural proteins (capsid [C], precursor membrane [prM] and envelope glycoprotein [E]) that form the viral particle and seven non-structural (NS) proteins (NS1, NS2A, NS2B, NS3, NS4A, NS4B, and NS5) that play different roles in the viral replication cycle [1]. In the case of HCV, the core (C) and glycoproteins E1 and E2 form the structural components of the virion, with p7, NS2, NS3, NS4A, NS4B, NS5A, and NS5B making the non-structural proteins [4]. Importantly, as the major virion surface protein, the viral E protein is the main target of neutralizing antibody response, and hence E antigens are largely found as immunogens in all approved and most developing Flaviviridae vaccine candidates [5]. Amongst the aforementioned viruses, YFV, JEV, DENV, and TBEV are currently the only ones with vaccines licensed for human use [5]. While the licensed YFV, JEV, and TBEV vaccines have demonstrated longstanding protective efficacy [5], the recently licensed DENV vaccine, however, is found to be detrimental to non-infected individuals, and only exerts protection selectively in individuals who have been previously infected [6], hence warranting new and better vaccines. The development of effective vaccines against Flaviviridae viruses continues to be fraught with difficulties due to their stringent requirements of their safety and immunogenicity. All forms of vaccine platforms, namely purified inactivated vaccine, subunit (DNA, mRNA, peptide, viral vector, E protein), virus-like particle (VLP) vaccine, and live-attenuated (native virus, chimeric flavivirus) vaccine, have been utilized in the hope of overcoming the challenges of producing ideally safe and efficacious vaccines that are cost-effective, particularly for viruses that currently have no existing licensed vaccines.

While most vaccines approved for human use against Flaviviridae viruses, and many vaccine candidates in ongoing development and trials, are based on purified inactivated and live inactivated vaccines, which can elicit good immunogenicity, these vaccines are generally limited by safety concerns over incomplete inactivation and the risk of infectious attenuated virus reversion to a pathogenic state, respectively [7]. In comparison, non-infectious subunit vaccines possess superior safety. Of which, the VLP vaccine platform possess additional unique advantageous qualities over purified inactivated and live-attenuated vaccines, and other subunit vaccine types, making it highly potential for development of successful Flaviviridae vaccines. The VLP vaccine platform is based on the simple expression of self-assembling viral structural proteins (prME or CprME for most members of the Flaviviridae family, and C, E1 and E2 for HCV) [7,8] to produce non-replicative VLPs lacking a viral genome but retaining the morphology and antigenicity of live infectious virions, thus rendering VLP vaccines highly safe but immunogenic [9]. Their unique feature of presenting viral antigens in highly native surface geometry organization and conformation allow VLP vaccines to efficiently elicit strong B-cell activation for high titre neutralizing antibody production, as well as activation of cellular immune response, thus inducing more potent protective immune responses as compared to subunit vaccines based on single proteins [9,10]. VLP vaccines also present significant advantages with respect to their low cost and ease of production, mainly requiring relatively inexpensive stable mammalian, insect, plant, yeast, or bacteria producer cell lines as the VLP expression system [9]. Finally, VLP vaccines have achieved successful protection against hepatitis B virus (HBV), human papilloma virus, hepatitis E virus, and influenza virus, and are licensed for use in humans [7], supporting its suitability as an effective vaccination strategy. Here in this review, we focus on the potential of the VLP vaccine platform in the current vaccine development against the different species of Flaviviridae viruses named above.

## 2. Yellow Fever Virus

Yellow fever virus (YFV) is a mosquito-borne flavivirus transmitted through *Aedes* or *Haemagogus* mosquito species, and the virus is endemic to Africa and South America with some reports in North America and Europe as well [11,12]. While the virus has yet to be documented in Asia or Oceania, the *Aedes* mosquito species can be found in these continents—indicating a possibility of future infection [13]. In fact, research regarding the susceptibility of these regions to YFV infection for Indonesia, Malaysia, and Thailand were found to be high [14]. First discovered around 1648 in Mexico and then first isolated in 1927, there are still ongoing outbreaks today with similar symptoms to other flaviviruses: fever, chills, myalgia, nausea, headaches, and/or fatigue in most cases. Nevertheless, the possibility of neurological complications and long-lasting neurological sequelae is an ever-present danger [11,15,16,17]. Available since the 1930s, the YFV 17D vaccine is regarded as one of the safest and most effective live-attenuated viral vaccines ever developed, with >99% of vaccinated people developing protective antibodies within a month and conferring immunity for over 30 years to life [11,15,18]. However, with YFV outbreaks still occurring and causing high mortality rates, as well as a depletion of the world’s emergency 17D vaccine stockpile reserve [19], an urgent need for the development of new alternative YFV vaccines is thus created.

While scientists tried to create a Vero cell-derived inactivated vaccine, it inevitably failed after phase I due to the lack of participants in the study [5]. The newest vaccination approach is DNA-launched live-attenuated vaccines that feature genetic stability, high purity, and a simpler means of production [20], yet they are still associated with safety concerns due to their ability to mutate and cause severe adverse events post-vaccination ranging from organ system failure to death [5]. Limited information is available regarding VLP vaccine candidates against YFV, but in 2006 and 2007 a group described the production of replication-defective pseudoinfectious YFV particles (characterized by capsid-deleted genome), that can in turn undergo a single round of infection in vitro and in vivo, to produce subviral particles (lacking nucleocapsid and viral genome), which present the immunogenic YFV E protein to elicit high-titre neutralizing antibodies and complete protection against viral challenges [21,22]. The authors also demonstrated that the pseudoinfectious YFV particles are highly safe in baby and adult mice [21]. Although the YFV pseudoparticles differ from VLPs in the presence of a viral subgenome, the subsequent mechanism of immunization achieved by the subviral particles formed after infection with the pseudoinfectious YFV particles is highly similar to how the VLP vaccine strategy works. The potential of the YFV pseudoinfectious particles to serve as YFV vaccine thus has positive implications on the suitability of the closely similar VLP vaccine platform for the development of effective YFV vaccines. Julander et al., on the other hand, developed a modified vaccinia virus Ankara (MVA)-based YFV vaccine (MVA-BN-YF), which is a non-replicating vector that expresses the prM and E proteins of the YFV 17D strain [23]. MVA-BN-YF induced neutralizing antibody production in the vaccinated hamsters, providing protection against YFV challenge [23]. Although the results have not been published, a phase I clinical trial of MVA-BN-YF was completed in 2018 (NCT02743455).

## 3. West Nile Virus

The West Nile virus (WNV) is a re-emerging virus that was first detected in 1937 in the West Nile district in Uganda [24]. Transmitted by *Culex* mosquitoes, WNV typically infects birds and animals with humans serving as dead-end hosts. Human infection is usually asymptomatic or associated with mild self-limiting symptoms. However, serious neurological complications (encephalitis) and death have been associated with major WNV outbreaks [25]. Although several veterinary WNV vaccines, including inactivated whole WNV vaccines [26], a recombinant vector-based vaccine expressing WNV prM and E proteins [27], and a chimeric YFV backbone-based vaccine pseudo-typed with WNV prM and E proteins [28], are available, there is currently no protective human vaccine against this virus. Due to the success of the aforementioned vaccines in animals, similar strategies have been replicated for human vaccines, with some currently in clinical trials. However, most of the vaccines undergoing clinical trials require multiple doses to elicit a protective immune response [29]. Furthermore, given that WNV mostly infects the elderly and immunocompromised people [30], the safety profile of these vaccines in such populations is a cause for concern. Thus, the continuous identification of safer and effective vaccines against WNV is highly desirable.

Owing to its safety and immunogenicity in animal models, several groups have explored the use of VLP as vaccine candidates against WNV. Qiao et al. in 2004 engineered and produced WNV prME or CprME expressing VLPs using a recombinant baculovirus expression system in Sf9 insect cell line [31]. Immunization of BALB/c mice with the purified prME WNV VLPs induced WNV E-specific neutralizing antibodies, leading to sterilizing immunity and complete protection with no detectable viremia and viral RNA in mice brains, and could thus potentially serve as a safe and effective vaccine option in humans. Following the above discovery, in 2010, Spohn et al. generated a conjugate WNV VLP (DIII-C-AP205) vaccine expressing the immunogenic domain III of WNV E protein on the surface of the AP205 bacteriophage, the coat protein of which is known to assemble into VLPs upon expression in *E. coli* [32]. Vaccination of mice with the DIII-C-AP205 conjugate vaccine led to the production of DIII-specific immunoglobulin G (IgG) antibody response after a single subcutaneous immunization, which protected them from a lethal WNV strain NY99 challenge. Also in 2010, using a different approach than before, Ohtaki and colleagues produced WNV prME VLPs by establishing CHO cells clones that stably secrete the VLPs by transfecting the cells with an expression vector encoding the WNV prME proteins followed by antibiotic selection [33]. They produced two types of VLPs—fast sedimenting VLP (F-VLPs) which express mature M and E proteins and slow sedimenting VLP (S-VLPs) consisting of E protein and immature prM protein. The immunization of mice with the F-VLP was more effective in inducing neutralizing antibody production and protecting mice from a lethal WNV challenge than the S-VLP, suggesting that the proper processing and conformational organization of these surface glycoproteins are important for their immunogenicity.

Finally, in a recent study, Taylor et al. produced WNV VLPs using a replication-defective herpes simplex virus 1 (HSV-1) recombinant vector and showed that immunization of mice with these recombinant VLPs elicited a specific anti-WNV IgG response [34]. Together, these results demonstrate that WNV VLPs effectively elicit neutralizing antibody response in mice which is protective against lethal WNV challenge.

## 4. Japanese Encephalitis Virus

Japanese encephalitis virus (JEV), an arbovirus transmitted by *Culex* mosquitoes and belonging to the flavivirus genus, is known as an important cause of viral encephalitis in humans, and its infection is associated with high mortality. Besides, almost half of the patients who survived JEV infection develop serious neurological sequelae. Although the current existing vaccines, including the live-attenuated and Vero cell-derived inactivated vaccines acquired from JEV genotype (G) 3, are effective against the virus [35], these vaccines are associated with allergic reactions in some cases. Moreover, a recent study found that immunization with the current JEV G3-based vaccines induced low levels of neutralizing/protective antibodies against the emerging JEV G5 virus [36]. These findings underscore the need to further identify safer and more effective vaccination strategies against JEV.

Like other viruses in the Flaviviridae family, several groups have explored the use of VLPs as vaccine candidates against JEV, with the earliest study in 1992 by Konishi and colleagues. These authors engineered a recombinant vaccinia virus encoding the immunogenic prME genes of JEV, the expression of which in Hela cells led to the production of JEV prME VLPs. In two separate studies, they showed that the recombinant vaccinia virus-derived JEV prME VLPs are highly immunogenic in inducing production of both JEV-specific neutralizing antibodies and T cell responses in mice with or without the presence of an adjuvant [37,38]. Although viral-based vectors are very effective at gene delivery, an inherent problem associated with these vectors is the presence of infectious particles. This issue can be circumvented by using alternative means of gene delivery such as plasmid DNAs. In an effort to produce safer JEV VLPs devoid of viral contaminants, Konishi et al. stably transfected CHO-K1 cells with a plasmid encoding JEV E and a mutated prM which prevents its cleavage by the furin protease, allowing these cells to continuously secrete large amounts of JEV VLPs without viral contamination and at the same minimizing cytotoxicity to the producer cells [39]. Immunization of mice with the mutant VLP elicited similar immunogenicity and protection to those with the native cleavage site. Similar immunogenicity/protection in mice and pigs was respectively observed when plasmids encoding JEV prME genes were stably expressed in rabbit kidney-derived RK13 and CHO cells [40,41]. Following the above discoveries, several other groups also expressed plasmid vectors encoding JEV prME genes to produce JEV VLPs in different mammalian cells, yielding similar immunogenicity/protection in mice [42,43]. On the other hand, many groups have also successfully used the insect cell expression system to generate JEV VLPs [44,45,46,47,48]. However, the immunogenicity of the insect cell-derived JEV VLPs has not been generally tested in animals, except for the study by Matsuda et al. in which silkworm-derived JEV VLPs were shown to be immunogenic in rabbits [48].

## 5. Hepatitis C Virus

With 170 million people infected and over 175,000 new cases globally [49], hepatitis C virus (HCV) remains a major important public health concern. Currently, there is no effective vaccine against the virus, and although the introduction of direct-acting antivirals (DAAs) has remarkably improved HCV treatment, these drugs do not confer sterilizing immunity and thus cannot prevent reinfection after viral clearance. Therefore, using DAAs alone to control the HCV pandemic is unrealistic. To decrease the global burden of HCV infection by 2030 as envisioned by the World Health Organization (WHO), the development of a protective vaccine against this important human pathogen should be prioritized. Designing/developing effective vaccines against HCV requires a greater understanding of the immune response against the virus. Studies in patients who cleared the virus suggest that eliciting both humoral and cellular immune responses is crucial to clear the virus [50]. These findings suggest that an effective HCV vaccine should ideally induce both antibody and T cell responses. In contrast to the traditional approaches for vaccine development such as live-attenuated vaccines or inactivated vaccines which are met with limited success in generating vaccines for HCV, VLP vaccines can trigger the production of broadly neutralizing antibodies and/or T cell responses (CD4^+^ and CD8^+^ T cells), both of which are critical for HCV clearance.

HCV VLPs (containing C, E1, and E2) were first produced in 1998 by Baumert et al. via a recombinant baculovirus expression system in Sf9 insect cells, generating HCV VLPs which displayed similar morphological, biophysical, and post-translational modification patterns (acetylation and glycosylation) to the authentic VLPs produced in mammalian cells [8]. This finding supplied the first evidence that HCV structural proteins alone expressed in insect cells can assemble into VLPs, and thus provided the impetus for using VLPs as vaccine candidates against HCV. The authors also showed that the insect cell-derived HCV VLPs reacted with sera of persons infected with various HCV genotypes, suggesting that HCV VLPs may contain epitopes that are conserved across all strains. Furthermore, the intraperitoneal immunization of mice resulted in the induction of a high-titer antibody response against the core and E2 proteins, as well as virus-specific T cells responses (CD4^+^ and CD8^+^ T cell responses), and interferon-gamma (IFN-γ) production [51,52]. In another study, vaccination of chimpanzees with insect cell-derived HCV VLPs predominantly induced the production of HCV-specific cellular immune response which partially protected these animals from HCV challenge (low viremia in the immunized group vs. high viremia in the control group). However, there was no detectable antibody response against the HCV structural proteins (C, E1, and E2) even after repeated immunization, suggesting that the HCV VLPs do not trigger antibody response in chimpanzees [53]. The reason why the HCV VLPs induced an antibody response in mice but not in chimpanzees is unclear, but could be due to an inter-species difference in immune response to the VLPs. Furthermore, the source of VLPs can shape the type of immune response in chimpanzees. Although VLP production in insect cells is attractive, in part, due to rapid protein expression and the induction of eukaryotic cell-like post-translational modifications (PTMs), the PTM in these cells is not as complete as those in the eukaryotic cells. In fact, it has been reported that the vaccination of chimpanzees with recombinant HCV proteins expressed in mammalian cells, but not those expressed in insect/yeast cells, protect the chimps from viral challenges [54]. This finding underscores the importance of the expression system used for producing HCV VLPs. Nonetheless, the above findings suggest that insect cell-derived VLPs are immunogenic and could serve as good vaccine candidates against HCV.

Using a similar approach (adenovirus carrying HCV structural proteins C, E1, and E2), but with the aim of improving the immunogenicity of the VLPs, Chua et al. in 2012 demonstrated that co-formulation of the HCV VLPs with the anionic self-adjuvanting lipopeptide containing the Toll-like receptor 2 (TLR2) agonist Pam_2_Cys (E_8_Pam_2_Cys) enhanced the immunogenicity of the Huh-7 hepatoma cells-derived HCV VLPs. Specifically, the vaccination of mice with the VLP+E_8_Pam_2_Cys resulted in better VLP and E2-specific antibody responses than the non-adjuvanted VLPs. In addition, mice vaccinated with VLP+E_8_Pam_2_Cys induced dendritic cell (DC) maturation and, in case of the HLA2.1 transgenic mice, produced better VLP-specific IFN-γ-mediated responses compared to the non-adjuvanted VLPs [55]. The fact that the adjuvanted VLPs induced DC maturation is promising, given the role of DCs in directing the adaptive immune response to vaccines [56]. In a similar study but without adjuvants, the above authors demonstrated that hepatocyte-derived HCV VLPs could elicit both humoral and CD8^+^ T cell responses in mice [57]. To extend the above findings and produce broader immune responses, Christiansen et al. developed a quadrivalent HCV VLP vaccine against genotypes 1a/1b/2/3a. Immunization of mice and pigs with this vaccine induced the production of neutralizing antibody responses coupled with strong B and T cell responses. Importantly, these quadrivalent HCV VLPs bound strongly to the selective human monoclonal antibodies which target the conserved domain B and D epitopes of the E2 protein, indicating that the VLPs express these critical regions on its surface and that antibodies raised against them could produce broad cross-reactive neutralizing antibody responses [58,59]. Using a different approach, Beaumont et al. constructed chimeric HBV-HCV envelope proteins consisting of the HBV S protein, the N-terminal of which was replaced with HCV envelope proteins E1 or E2. These chimeric HBV-HCV proteins co-assembled with the wild type S protein to produce subviral particles in CHO cells [60,61]. The immunization of rabbits with the purified chimeric VLPs induced specific antibody responses against both HBV and HCV envelope proteins, and sera containing these bivalent chimeric VLPs neutralized various heterologous cell culture-derived HCV strains including genotypes 1a, 1b, 2a, and 3, suggesting that these antibodies could be broadly neutralizing [61]. Interestingly, in a follow-up study, the authors demonstrated that animals vaccinated with chimeric VLPs containing E1 and E2 separately yielded better E1- and E2-specific antibody responses than those immunized with the VLPs encoding the E1E2 heterodimer. Further, the anti-E1 and -E2 antibodies displayed additive cross-neutralization effects against HCV genotypes, highlighting the significance of using E1 and E2 separately as immunogens to produce more effective vaccines [62]. Although the vaccination of animals with these chimeric VLPs induced the production of broadly cross-reactive neutralizing antibodies, whether or not these VLPs can trigger the induction of the cellular immune responses was not investigated. Considering the importance of cellular immunity in HCV vaccine development, it will be interesting to determine whether these vaccines can, in addition to triggering broad cross-reactive neutralizing antibodies, also induce a cellular immune response in vivo.

Retroviral expression vectors are useful for stable gene expression and have also been explored for HCV vaccine development. In 2009, Desjardin and colleagues generated HCV pseudoparticles (HCVpp) containing HCV-E1 and E2 that assembled on a retrovirus core derived from murine leukemia virus (MLV). Specifically, the authors constructed plasmid DNAs encoding the HCV E1E2 glycoproteins and the gag/pol protein of MLV to generate plasmo-retroVLP^E1E2^, expression of which in vivo produces HCVpp [63]. Due to the importance of recombinant adenovirus 5 (rAd5)-based vectors in eliciting a strong antigen-specific immune response, these authors also generated rAd5 encoding the HCV E1E2 proteins (rAd5^E1E2^), which was used to prime mice before boosting with plasmo-retroVLP^E1E2^. Results show that the plasmo-retroVLP^E1E2^ boost significantly enhanced the E1E2-specific T cell responses as well as the antibody response against E2 in mice. In contrast, boosting with the control plasmo-retroVLP^E1E2^ that cannot assemble into HCVpp did not affect the immune response, suggesting that the particulate forms of E1 and E2 are essential for eliciting a more efficient immune response. Subsequently, the authors tested the efficacy of the prime-boost immunization strategy on macaques [64]. Priming macaques with rAd5^E1E2^ resulted in similar T cell responses against E1 and E2, with E2 specific antibody response but no E1 antibody response, suggesting that E2 is immuno-dominant over E1. Boosting with plasmo-retroVLP^E1E2^ triggered the induction of E1-specific antibodies and increased the anti-E2 antibody response, in addition to generating neutralizing antibody responses that neutralized several HCV genotypes, including genotypes 1b, 2a, 2b, 4, and 5 [64]. These findings highlight the efficacy of the prime-boost strategy using retroviral vectors to generate both humoral and adaptive responses in animals.

## 6. Dengue Virus

Commonly known due to its highly prevalent status as a widespread arbovirus that is endemic in over 125 countries in tropical and subtropical regions of the world, dengue virus (DENV) is recognised by the World Health Organisation (WHO) as the most important mosquito-borne viral disease transmitted by *Aedes* mosquitoes, mainly *Aedes aegypti*. While the majority of DENV infection cases end in mild, febrile, self-limiting disease, DENV is also known to cause severe diseases called dengue hemorrhagic fever (DHF) or dengue shock syndrome (DSS), characterized by vascular leakage, bleeding disorder, and shock that can lead to death [6]. With global estimates of up to 400 million dengue infections, 500,000 incidences of severe dengue disease (DHF/DSS) and over 20,000 dengue related deaths yearly [6,65], DENV imposes a significant public health concern and socioeconomic burden worldwide. Additionally, as DENV is expected to continue its geographical expansion due to factors including climate change and increased travel, DENV vaccination is of great importance for the prevention and control of its disease. However, as DENV circulates as four serotypes DENV1-4, and with the apparent hypothesized effect of antibody-dependent enhancement (ADE) in DENV infection, for 70 years, DENV vaccine development has been difficult and unsuccessful due to the requirement that an effective vaccine should be tetravalent that is equally protective against each of the four serotypes to avoid the risk of ADE of DENV infection [6,66].

Initially conceptualized by Scott Halstead in the context of DENV infection, ADE describes the phenomenon where pre-existing protective DENV serotype-specific antibodies are cross-reactive but not cross-protective against a different serotype DENV, and thus facilitate heterologous DENV Fc-receptor-mediated infectivity instead into myeloid cells such as monocytes and macrophages, leading to increased viral load and development of the severe and potentially fatal DHF/DSS [6]. The occurrence of ADE has been a major concern with DENV vaccination due to fears that immunization may instead predispose dengue-naïve individuals to the severe outcomes of DENV infection. Hence, the avoidance of ADE constitutes a main key criterion in the development of a successful vaccine that is protective against DENV without inducing an increased risk of infection mediated by ADE. Additionally, in recent years with increasing co-circulation of the antigenically related Zika (ZIKV) flavivirus, the speculations about DENV ADE have also been extrapolated to the context of ZIKV infection, and vice versa intuitively. Indeed, the cross-reactivity of DENV antibodies to ZIKV has been reported [67], which could potentially promote the ADE of ZIKV infection, thus further complicating the development of an effectively protective DENV vaccine.

To date, Dengvaxia, a live-attenuated chimeric yellow fever/dengue tetravalent dengue vaccine (CYD-TDV) developed by Sanofi Pasteur is the first and only licensed DENV vaccine since 2015. Currently, it is licensed for use in 20 dengue-endemic countries (with seroprevalence of 70% and above) in Asia, Latin America, and Australia for persons aged 9–45 [6,68]. However, the ongoing surveillance of its efficacy up until recent post-hoc studies performed in 2017 revealed major caveats of the vaccine as it is found to be poorly protective against DENV2, and indeed increases the risk of development of severe dengue disease in dengue-naïve seronegative individuals [6,68]. As such, the WHO recommends provision of the vaccine only to DENV seropositive people for which it has proved to be safe and effective, necessitating a pre-vaccination serostatus screening [6]. Dengvaxia is therefore also contraindicated as a travellers’ vaccine for travellers from non-DENV endemic countries as they are likely dengue seronegative [68]. Due to the significant limitations on the usage of Dengvaxia, many other tetravalent DENV vaccine candidates, including VLP vaccines, are being extensively studied and tested in the hope of finding another effective vaccine. In the past two decades, a number of DENV VLPs have been evaluated as vaccine candidates [69,70,71,72,73,74,75,76,77,78,79,80,81,82,83,84]. Of which, tetravalent DENV VLP vaccines, recently developed and currently in preclinical testing, possess several features that confer safety, immunogenicity, and production advantages that ideally fulfilled the requirements for a successful DENV vaccine development [81,82,83,84]. Generated by the co-expression of all four DENV serotypes’ envelope proteins, these VLPs have mosaic tetravalent constructs, as opposed to many live-attenuated vaccine candidates that are mixture of monovalent vaccines against each of the four serotypes. Such a mosaic VLP vaccine candidate has a cost-saving advantage over the need to produce and purify four monovalent E VLPs for a tetravalent mixture [84]. Furthermore, notably, some of these VLPs lack the viral prM protein in their structure, which circumvents the induction of cross-reactive non-neutralizing anti-prM antibodies that mediate ADE [84]. The VLP-induced neutralizing antibodies are predominantly directed against DENV envelope domain III (EDIII), and are shown not to cause ADE in vivo [81,82,83,84]. This feature which avoids the risk of ADE is highly desirable for DENV vaccine, and further enhances the safety profile of the non-infectious VLP vaccine candidates. These favourable attributes strongly support the VLP vaccine platform as excellent candidates for the development of an effective DENV vaccine that is safe for young children and seronegative individuals, and neutralizing against all DENV serotypes without the risk of ADE [6].

## 7. Zika Virus

Though first identified from Uganda’s Zika forest in 1940s, Zika virus (ZIKV) only recently made its name as an emerging infectious virus almost 70 years later with its explosive 2015 outbreak in the Americas [85]. As an arbovirus like DENV, ZIKV is primarily transmitted to humans via the bites of infected female *Aedes* mosquitoes, particularly *Aedes aegypti* [85]. In addition, non-vector-borne transmissions in humans occur via laboratory-acquired infections, nosocomial infections, sexual transmission, maternofetal transmission, and transfusion/transplant-transmitted infections [85]. Despite a history of innocuous spread in Africa and Asia throughout 1950s to the 1990s, ZIKV broke silent circulation with its first large epidemic on the Western Pacific Island of Yap in 2007, infecting almost 75% of the population and causing ~900 symptomatic cases [86]. Subsequently, in 2013–2015, more Pacific islands became affected, recording a second greater epidemic with 30,000 symptomatic cases in French Polynesia [85]. Soon after in May 2015, ZIKV’s inaugural emergence in Brazil marked its third major epidemic, affecting over 20 regions in the Americas and the Caribbean by the end of January 2016 [85]. Also in 2015, ZIKV re-emergence in the Cape Verde Islands in Africa sparked an epidemic, deviating from its history of sporadic infections [85].

Currently, while ZIKV is no longer a Public Health Emergency of International Concern (PHEIC), it continues to pose a vicious threat to global health security with autochthonous transmission in reportedly 87 countries and territories according to WHO [87]. Moreover, although symptomatic Zika fever is mostly mild and self-limiting with flu-like symptoms, its association with severe neurological sequelae such as Guillain–Barré syndrome (GBS), acute myelitis, encephalitis in the Pacific Islands, and especially infant microcephaly in the Americas in the recent ZIKV emergence [85] has incited global concern. Additionally, ZIKV’s pandemic potential is precariously anticipated due to speculations of its continued global spread to follow that of the widespread DENV, since ZIKV shares the same epidemiology and mosquito vectors, as well as facilitation of its spread by increasing travel of infected individuals especially to tropical regions lacking mosquito control and herd immunity to ZIKV [85]. A total of 61 regions with abundant competent *Aedes* mosquito-vectors, and thus at risk of future ZIKV emergence, have been identified [87]. In the face of the ongoing ZIKV pandemic with no licensed vaccine nor approved specific antiviral drugs [85], development of effective vaccination is highly pursued as a crucial prevention and control of ZIKV infection, with particular importance for pregnant women given the high incidence of severe ZIKV induced foetal birth defects collectively termed ‘congenital Zika syndrome’ (CZS).

Of over 45 ZIKV vaccine candidates evaluated since 2015, at least nine are in phase I or II clinical trials [88,89], and recent development included several Zika VLP vaccines mostly in early research stages and preclinical animal studies [90]. From the cumulative preclinical and clinical trial results from different ZIKV vaccine candidates, it has been primarily established that neutralizing antibodies production strongly correlates with protective efficacy [88], and is apparently sufficient for complete protection in both mice and rhesus monkey models [91,92,93]. As the earliest developed ZIKV vaccine platforms, purified-inactivated vaccine and DNA vaccine have generally demonstrated good safety and immunogenicity profiles in inducing neutralizing ZIKV antibodies production [94,95,96]. However, the newer VLP vaccine candidates possess relative advantages in their safety and immunogenicity that render VLP vaccine a valuable platform for ZIKV vaccine development. With a primary goal in preventing CZS, pregnant women are a priority target population for vaccination against ZIKV, making safety a top criterion required of a successful ZIKV vaccine. As such, firstly, being non-infectious, the VLP vaccine platform has superior safety feature favourable over live-attenuated vaccine candidates against ZIKV. Secondly, as compared to other non-infectious vaccine candidates, most commonly purified-inactivated and DNA vaccines against ZIKV, VLP vaccines generated in mammalian cells have demonstrated greater immunogenicity profiles in separate studies published in 2017. Namely, ZIKV prME and CprME VLPs were developed and their vaccination efficacies were evaluated against ZIKV prME and CprME DNA vaccination as well as formalin inactivated ZIKV vaccination by Garg et al. [97] and Boigard et al. [98], respectively. ZIKV CprME VLPs were found to be more immunogenic than prME VLPs [97], and both studies reported superior ZIKV neutralizing antibody titers induced in mice by ZIKV CprME VLPs in comparison to the analogous DNA and purified-inactivated vaccines [97,98]. Later in 2018, Dai et al. developed a baculovirus-expressed prME VLP and demonstrated its ability to induce neutralizing antibodies and memory T cell response in mice [99]; however, in contrast to the previous two VLPs, the immune responses triggered by the baculovirus-expressed VLP appeared to be weaker than those induced by purified-inactivated vaccine.

Besides the aforementioned ZIKV prME and CprME VLPs, other VLP variations were also generated and evaluated. A VLP displaying ZIKV E protein domain III on hepatitis B core antigen and produced in the *Nicotiana benthamiana* plant expression system is reported to induce strong humoral as well as cellular immune protection in mice against various ZIKV strains, without causing antibody-dependent enhancement of DENV infection in Fcγ receptor-expressing cells [100]. Another chimeric VLP system using the replication-deficient chimpanzee adenoviral (ChAdOx1) vector to express ZIKV prM and E proteins [101] was found to trigger both humoral and cellular immune responses and prevent viremia, with the absence of an envelope transmembrane domain resulting in better immunogenicity. The recombinant ChAdOx1 ZIKV is being evaluated in a phase I clinical trial (NCT04015648). These important findings further promote the potential of the VLP vaccine platform as a cost-effective, highly protective and safe ZIKV vaccination strategy, especially as cellular immunity has recently shown to play protective role against ZIKV burden in mice [102], and the possibility of cross-reactive antibody-induced enhancement of DENV infection is a key concern with ZIKV vaccination.

## 8. Tick-Borne Encephalitis Virus and Powassan Virus

Emerging viral infections cover viruses that have emerged at the turn of the century or viruses that have existed but have recently seen an uptick in documented cases. Of these new emerging viral infections, tick-borne encephalitis virus (TBEV) and Powassan virus (POWV) are two viruses from the *Flaviviridae* family tick-borne encephalitis complex, a group of encephalitis-associated viruses that share substantial similarity in their genome and amino acid sequences [103]. Both TBEV and POWV are transmitted through ticks from the *Ixodes* genus—however, whereas TBEV is more prevalent across the globe, POWV is isolated to North America. TBEV and POWV both have incubation periods after tick bites but can be asymptomatic. In TBEV, the second phase of disease is what causes morbidity and mortality with meningitis and meningoencephalitis being most prevalent with long-term sequelae affecting patients after infection [104]. To date, the tick-borne encephalitis complex has a number of licensed vaccines that are protective against all three serotypes of TBEV circulating in Europe and Asia, but no drug with demonstrated efficacy [105].

Of the current TBEV vaccination strategies, all of them include formalin-inactivation after infecting chicken embryo cells with various European TBEV strains; however, these vaccines are usually expensive, require multiple doses to be effective, all require boosters, and are poor in immunogenicity in comparison to live-attenuated vaccines [105,106]. Before these vaccines were available, there were a few alternatives in trial, but were discontinued due to neurological complications [107]. Even with the current vaccination possibilities, there are still reported cases of vaccinated individuals who still become infected with tick-borne encephalitis. Of these, anti-POWV titers from serum taken from vaccinated individuals are lower because of less amino acid conservation across the glycoprotein genomes between POWV and TBEV [108]. These signal a need for a more effective and comprehensive vaccination strategy. The most recent development in POWV vaccinations include a lipid nanoparticle (LNP) encapsulated mRNA. The mRNA vaccine encodes two important genes: prM and E that theoretically would self-assemble into a viable particle. After injection, the LNPs create subviral particles (SVPs) while the modified mRNA enhances helper T cell and B cell responses. As a result, inoculated mice produced neutralizing antibodies towards POWV and even TBEV with little amino acid conservation [109]. As for TBEV, several approaches have been used to generate VLPs as an antigen [110,111,112,113,114,115,116], yet none of these VLPs were examined as a vaccine candidate in animal models. In the next few years the use of these new vaccination strategies will be in the aims of preventing less infections in vaccinated individuals, establishing a vaccine that works on a wide variety of flaviviral TBE complex, and creating vaccines that are cost-effective and confer long-lasting immunity.

## 9. Discussion and Conclusions

In summary, given the favourable preclinical safety, immunogenicity profiles, and relatively simple and low cost production, as demonstrated by the various VLP vaccine candidates reviewed above, the VLP vaccine platform has strong potential as an effective approach in the current vaccine development efforts against the different Flaviviridae viruses. Although mostly in early research and preclinical studies presently, positive reports of their protective efficacies in animal models warrant further development and evaluation of the different VLP vaccine candidates, especially for WNV, HCV, DENV, and ZIKV, which still lack highly protective successful vaccines.

Nonetheless, like the other purified inactivated vaccines, live-attenuated vaccines, and subunit vaccines, the VLP vaccine platform is not without limitations. While multiple studies have reported effective viral neutralization and complete protective efficacy from single VLP vaccine immunization against WNV, JEV, HCV, DENV, and ZIKV, the induction of lasting sterilizing immunity and the optimal immunization regime required remain to be determined. Due to its non-infectious nature, it is safe and thus preferred due to suitability for larger target populations, including pregnant women (particularly important for ZIKV vaccine), immunocompromised infants, as well as young and senior individuals, but single-dose vaccination efficacy (usually seen with live-attenuated vaccines) is unlikely. Multiple dosing and periodic boosting are commonly required for non-infectious vaccines, which can raise the cost of vaccination as well as the challenge for immunizing remote populations [88]. Besides these anticipated limitations, it should also be noted that the type of expression system used has important influences on the performance and production of the VLP vaccine, and thus should be carefully chosen considering the specific virus of interest. Over 170 different prokaryote (bacteria) and eukaryote (yeast, plant, insect, mammalian) expression host systems can be used to produce VLPs [9]. The levels of VLP expression and secretion vary depending on the virus type and expression system used. Generally, in terms of production yield, bacteria expression systems are known to be more efficient than eukaryote expression hosts [9], and amongst the eukaryotes, insect and yeast expression systems usually produce greater levels of VLPs [7]. However, mammalian expression hosts, particularly human cells, are usually the most ideal for proper post-translational modifications required for correct viral prME proteins folding, glycosylation and maturation, and subsequent VLP assembly and secretion [7]. In view of this, strategies such as enhancing VLP assembly by co-expressing efficient prM cleavage proteases, and/or enhancing VLP secretion by using appropriate native signal sequences of the expression cell type chosen, can be adopted to optimize VLP production efficiency [7]. Adjustments in producer cell culture conditions (e.g., temperature, pH), and prME genetic modifications can also be made to enrich the population of mature VLPs produced to maximise overall immunogenicity and thus protective efficacy of the VLP vaccine [7]. A summary of the strategies of the VLPs developed for vaccine candidates is listed below in Table 1.

Finally, based on the latest understanding of the key safety and immunogenicity characteristics required for an effective vaccine against each of the different Flaviviridae viruses, future VLP vaccine developments can place a greater focus on ensuring vaccine safety (avoidance of ADE for DENV) and the stimulation of T cell cellular immunity responses, which has new-found importance in contributing to the successful protection against Flaviviridae viruses including HCV, DENV, and ZIKV. 

## Figures and Tables

**Table 1:** Summary of flavivirus-like particles for vaccine development.

Virus (Strain)	Viral Proteins	Vector System	Producer Cell	Development Stage	Triggered Immunity	Ref.
YFV (17D)	prM and E	Capsid-deleted YFV	BHK-21	In vivo	Antibody	[21,22]
YFV (17D)	prM and E	MVA-BN	Primary chicken embryo fibroblast	Phase I clinical trial	Antibody	[23]
WNV (HNY1999)	C, prM, and E	Recombinant baculovirus	Sf9	In vivo	Antibody	[31]
WNV (NY99)	EDIII	Bacteriophage (AP205)	*E.coli*	In vivo	Antibody	[32]
WNV (NY99)	prM and E	Plasmid DNA	CHO	In vivo	Antibody	[33]
WNV	prM, and E	Recombinant HSV-1	E11	In vivo	Antibody	[34]
JEV (Nakayama)	prM and E	Recombinant vaccinia virus	Hela	In vivo	Antibody and T cells	[37,38]
JEV (Nakayama)	prM and E	Plasmid DNA	CHO-K1	In vivo	Antibody	[39]
JEV (Beijing)	prM and E	Plasmid DNA	RK13	In vivo	Antibody	[40]
JEV (YL2009-4; G1)	prM and E	Plasmid DNA	CHO	In vivo	Antibody	[41]
JEV (Nakayama)	prM and E	Plasmid DNA	COS-1	In vivo	Antibody	[42]
JEV (SA14-14-2)	prM and E	Plasmid DNA	BHK-21	In vivo	Antibody	[43]
JEV (SA14)	prM and E	Drosophila Expression System	S2	In vivo	Antibody	[44]
JEV (Nakayama)	prM and E	Recombinant baculovirus	Sf9	In vitro	Not tested	[45,46]
JEV (P3)	prM and E	Recombinant baculovirus	Sf9	In vitro	Not tested	[47]
JEV (Nakayama)	prM and E	Recombinant baculovirus	BM-N	In vivo	Antibody	[48]
HCV (GT 1b)	C, E1, and E2	Recombinant baculovirus	Sf9	In vitro	Not tested	[8]
HCV (GT 1b)	C, E1, and E2	Recombinant baculovirus	Sf9	In vivo	Antibody	[51]
HCV (GT 1b)	C, E1, and E2	Recombinant baculovirus	Sf9	In vivo	Antibody, CD4^+^ and CD8^+^ T cells	[52]
HCV (GT 1b)	C, E1, and E2	Recombinant baculovirus	Sf9	In vivo	CD4^+^ and CD8^+^ T cells	[53]
HCV (GT 1a)	C, E1, and E2	Recombinant adenovirus	Huh-7	In vivo	Antibody	[55]
HCV (GT 1a)	C, E1, and E2	Recombinant adenovirus	Huh-7	In vivo	Antibody and CD8^+^ T cell	[57]
HCV (GT 1a, 1b, 2a, and 3a)	C, E1, and E2	Recombinant adenovirus	Huh-7	In vivo	Antibody, CD4^+^ and CD8^+^ T cells	[58,59]
HCV (GT 1a)	E1 and E2; E1-HBsAg and E2-HBsAg	Lentiviral vector	CHO	In vivo	Antibody	[61,62]
HCV (GT 1a)	E1 and E2	Retroviral vector	293T	In vivo	Antibody and CD8^+^ T cell	[63,64]
DENV-1	C, prM, and E	Plasmid DNA	*P. pastoris*	In vivo	Antibody	[69]
DENV-2	prM and E	Plasmid DNA	*P. pastoris*	In vivo	Antibody	[70]
DENV-1; DENV-2; DENV-3; DENV-4	prM and E	Plasmid DNA	293T	In vivo	Antibody and IFN-γ	[71]
DENV-1	prM and E	Plasmid DNA	*P. pastoris*	In vivo	Antibody and T cell	[72]
DENV-2	HBcAg-EDIII	Plasmid DNA	*E. coli*	In vivo	Antibody	[73]
DENV-2	HBcAg-EDIII	Plasmid DNA	*P. pastoris*	In vivo	Antibody	[74]
DENV-2	EDIII	Plasmid DNA	C6/36	In vivo	Antibody	[75]
DENV-1; DENV-2; DENV-3; DENV-4	Ecto E	Plasmid DNA	*P. pastoris*	In vivo	Antibody	[76,77,78,79]
DENV-1/2 (Bivalent)	Ecto E	Plasmid DNA	*P. pastoris*	In vivo	Antibody	[80]
DENV-1/2/3/4 (Tetravalent)	prM and E	Plasmid DNA	*P. pastoris*	In vivo	Antibody, TNF-α and IL-10	[81]
DENV-1/2/3/4 (Tetravalent)	prM and E with F108A mutation	Plasmid DNA	FreeStyle 293F	In vivo	Antibody	[82]
DENV-1/2/3/4 (Tetravalent)	HBsAg and Ecto E	Plasmid DNA	*P. pastoris*	In vivo	Antibody	[83]
DENV-1/2/3/4 (Tetravalent)	Ecto E	Plasmid DNA	*P. pastoris*	In vivo	Antibody	[84]
ZIKV (Z1106033)	C, prM, and E; prM and E	Plasmid DNA	293T	In vivo	Antibody	[97]
ZIKV (H/PF/2013)	C, prM, and E	Plasmid DNA	Expi293	In vivo	Antibody	[98]
ZIKV (PRVABC59)	EDIII	Recombinant tobacco mosaic virus	*N. benthamiana*	In vivo	Antibody and IFN-γ	[100]
ZIKV (Z1106033)	C, prM, and E	Plasmid DNA	293T	In vivo	Antibody	[97]
ZIKV (Asian)	prM and E	Recombinant adenovirus	HEK293	Phase I clinical trial	Antibody and IFN-γ	[101]
TBEV	prM and E	Plasmid DNA	COS-1	In vitro	Not tested	[110,111]
TBEV	prM and E	Plasmid DNA	293T	In vitro	Not tested	[112]
TBEV (Neudoerfl)	prM and E	Plasmid DNA	CHO-ME	In vitro	Not tested	[113]
TBEV	prM and E	Recombinant baculovirus	Sf9	In vitro	Not tested	[114]
TBEV (KrM 93)	prM and E	Plasmid DNA	*P. pastoris*	In vitro	Not tested	[115]
TBEV (Torö-2003 Swedish)	C, prM, and E; prM and E	Plasmid DNA	COS-1 or BHK-21	In vitro	Not tested	[116]
